# Intensive care of the cancer patient: recent achievements and remaining challenges

**DOI:** 10.1186/2110-5820-1-5

**Published:** 2011-03-23

**Authors:** Elie Azoulay, Marcio Soares, Michael Darmon, Dominique Benoit, Stephen Pastores, Bekele Afessa

**Affiliations:** 1 AP-HP, Hôpital Saint-Louis, Medical ICU, Paris, France; 2 University Paris-7 Paris-Diderot, UFR de Médecine, 75010 Paris, France; 3 D'Or Institute for Research and Education, Rio de Janeiro, Brazil; 4 Postgraduate Program, Instituto Nacional de Câncer, Rio de Janeiro, Brazil; 5 Intensive Care Department, Hôpital de Bellevue and Saint-Etienne University, Saint-Etienne, France; 6 Intensive Care Department, Ghent University Hospital, Ghent University, Ghent, Belgium; 7 Department of Anesthesiology and Critical Care Medicine, Memorial Sloan-Kettering Cancer Center, 1275 York Avenue C1179, New York, NY 10065, USA; 8 Division of Pulmonary and Critical Care Medicine, Mayo Clinic, Rochester, MN, USA

## Abstract

A few decades have passed since intensive care unit (ICU) beds have been available for critically ill patients with cancer. Although the initial reports showed dismal prognosis, recent data suggest that an increased number of patients with solid and hematological malignancies benefit from intensive care support, with dramatically decreased mortality rates. Advances in the management of the underlying malignancies and support of organ dysfunctions have led to survival gains in patients with life-threatening complications from the malignancy itself, as well as infectious and toxic adverse effects related to the oncological treatments. In this review, we will appraise the prognostic factors and discuss the overall perspective related to the management of critically ill patients with cancer. The prognostic significance of certain factors has changed over time. For example, neutropenia or autologous bone marrow transplantation (BMT) have less adverse prognostic implications than two decades ago. Similarly, because hematologists and oncologists select patients for ICU admission based on the characteristics of the malignancy, the underlying malignancy rarely influences short-term survival after ICU admission. Since the recent data do not clearly support the benefit of ICU support to unselected critically ill allogeneic BMT recipients, more outcome research is needed in this subgroup. Because of the overall increased survival that has been reported in critically ill patients with cancer, we outline an easy-to-use and evidence-based ICU admission triage criteria that may help avoid depriving life support to patients with cancer who can benefit. Lastly, we propose a research agenda to address unanswered questions.

## Introduction

The number of patients living with cancer has been increasing steadily [1-3]. The ageing population, improved diagnostic tools for cancer, and decrease in cancer-related mortality have contributed to this increase. The age-adjusted invasive cancer incidence rate (95% confidence interval) in the United States is 533.8 (532.6-535.1) per 100,000 population [4]. More than 1.4 million people were projected to be diagnosed with cancer in the United States in 2009 [3]. In Europe, there were an estimated 3,191,600 cancer cases diagnosed and 1,703,000 deaths from cancer in 2006 [5]. In 2005, more than 100,000 cases of hematological malignancies were diagnosed in the United States and approximately 230,000 in Europe [4,6]. Intensive chemotherapy regimens [7] and the use of new and more targeted therapeutic drugs have resulted in high cancer cure rates. However, the treatment often leads to drug-related organ toxicities and increased susceptibility to infection [8,9]. As a consequence, intensivists are increasingly managing patients with cancer who are admitted to the intensive care unit (ICU) for organ dysfunction--chiefly respiratory failure, originating from infectious, malignant, or toxic complications [10,11]. Timely recognition and early ICU admission offer opportunities to prevent and manage life-threatening complications that are cancer-related, including tumor lysis syndrome [12], leukostasis [13], and macrophage activation syndrome [14]. Managing organ dysfunction in critically ill cancer patients requires specialized skills by the intensivist and close collaboration between the intensivist and oncologist.

Critically ill cancer patients have lower survival rates compared with patients without comorbidities. However, their in-hospital mortality rates are not higher compared with critically ill patients with other comorbidities, such as heart failure, liver cirrhosis, or other serious chronic diseases [15]. Recent studies have shown that a substantial survival rate can be achieved even in severely ill patients with cancer [16-18]. Healthcare providers and patients often discuss the merits of providing mechanical ventilation, vasoactive agents, renal replacement therapy, or other life-sustaining treatments in patients with cancer [19]. There also are unresolved questions about whether part or all of these supportive therapies can be simultaneously administered with cancer-specific treatments, including chemotherapy [20-22]. More recently, the lack of survival benefit in cancer patients admitted to the ICU with multiple organ failure [10,17] has raised concerns about the timing of ICU admission [11].

This is not a systematic review but a consensus opinion from experts who care for critically ill patients with cancer. We plea for the development and implementation of broader ICU admission policies. Future observational research will be required to assess the validity of our conclusions.

### Cancer patients requiring ICU support: the ten truths (Tables 1 and 2)

**Table 1:** Prognosis in cancer patients needing intensive care support: the ten truths

1. Short-term survival after critical care illness has improved
2. Classic predictors of mortality are no longer relevant
3. Clinicians' understanding of organ dysfunction has improved
4. Some subgroups of patients continue to have high and unchanged mortality
5. The typically used triage criteria for ICU admission are unreliable
6. Three days of ICU management is warranted before making a final decision (ICU trial)
7. Attempt should be made to find a balance between noninvasive treatments and avoiding delays in optimal therapies
8. Close relationship and collaboration need to be developed between intensivists and hematologist/oncologists to increase skills of all sides in the global management of cancer patients
9. Early admission to the ICU for cancer patients is recommended
10. Doing everything possible, even cancer chemotherapy, may improve outcome

**Table 2:** Recent ICU advances in the management of critically ill cancer patients

1. Less restrictive admission policies [17]
2. Use of noninvasive mechanical ventilation [23,48,49]
3. Diagnostic strategy in acute respiratory failure [40,47,50,60]
4. Prevention of tumor lysis syndrome [12,58,59]
5. Management of acute kidney injury [37-39]
6. Advances in antifungal agents [102]
7. Transfusion policies [103]
8. Recognizing drug-related organ toxicities [104-106]
9. Understanding organ dysfunction in macrophage activation syndrome [14]
10. Diagnostic strategy in neurological involvement [107]

#### Short-term survival after critical care illness improved

**Figure 1:**
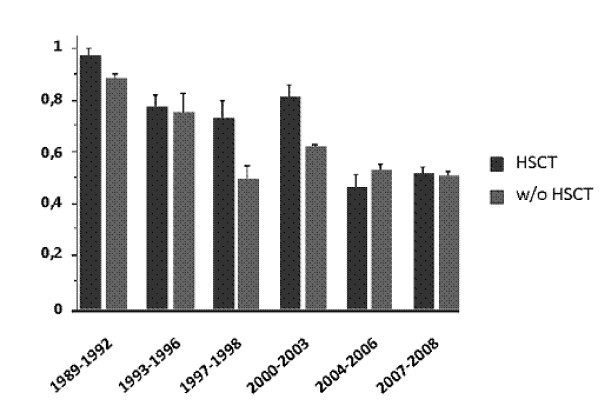
**Trends of mortality in critically ill cancer patients during the past two decades**. Unadjusted hospital mortality rates in critically ill cancer patients by year of study publication (clear gray). Unadjusted ICU mortality rates in bone marrow transplant recipients by year of study publication (dark gray)

Several studies comparing two time periods in subgroups of cancer patients have reported improvement in hospital survival during the past decade [10,11,23-28] (Figure 1). Recent prospective and retrospective cohort studies have documented lower mortality rates compared with the mortality rates previously reported [16,23,29-33]. Improved survival rates have been reported in cancer patients who require mechanical ventilation [23,30,34-36], renal replacement therapy [37-39], and vasopressors [26,27], as well as those with neurological complications [28]. The impact of the Surviving Sepsis Campaign may have played a part in the reduction in mortality seen during the past few years in patients with septic shock [26,27]. Although these studies include large cohorts of patients, most of them are retrospective and single-centered and do not provide adequate reasons for the improved survival. Only two prospective, multicenter, ICU outcome studies that involve cancer patients have been published to date [40]. Another limitation of these studies is the huge heterogeneity in patient case mix: medical and surgical patients; solid and hematologic cancer patients; allogeneic and autologous blood; and bone marrow transplant (BMT) recipients. Moreover, it is difficult to compare the results between the published studies because of the variations in ICU admission and discharge criteria as well as the settings and timing for the implementation of end-of-life decisions [41].

Although there may have been a general improvement in ICU mortality of cancer patients over time, five hypotheses have been proposed to account for the decreased mortality rate in these patients [19]: 1) an overall improved survival in cancer patients [42,43] related to the use of more intensive chemotherapeutic regimens [7], the development of more potent and targeted therapies [8,9,44,45], as well as advances in the supportive care and prevention of organ dysfunction [46]; 2) improved ICU management with the development of noninvasive diagnostic [40,47] and therapeutic strategies [23,48,49]; 3) ability to obtain the etiological diagnosis in patients with acute respiratory failure [40,49-51], e.g., bacterial infection [21,22,52]. In this regard, it is worth noting that patients admitted to ICUs with a large volume of hematological patients with acute respiratory failure experience a lower mortality [53]; 4) new strategies avoiding early chemotherapy during the course of chronic malignancies (such as watch-and-wait policies or immunotherapy) assist in managing patients with improved performance status with less organ-related toxicity and epithelial and endothelial dysfunction with its increased propensity for cardiovascular, renal, and pulmonary dysfunctions [54]; and 5) changes in triage patterns may have occurred that facilitate the ICU admission of cancer patients with the best chances for survival.

Overall, the medical literature has documented improvement in survival of critically ill patients with cancer. However, most of the studies have evaluated short-term outcomes, such as ICU, hospital, 28-day, and, rarely, 3- or 6-month survival. To our knowledge, no study has used long-term or meaningful outcomes, such as disease-free survival and quality of life after ICU admission.

#### Classic predictors of mortality are no longer relevant

Current studies still report that the need for mechanical ventilation, presence of invasive fungal infection, development of multiorgan failure, and high severity of illness scores are additional prognostic factors for mortality among cancer patients. Although these prognostic factors are important, they often are unreliable and mostly derived from inconsistent results. For example, a British multicenter study of patients with hematologic malignancy admitted to the ICU showed that BMT was a risk factor for increased hospital death [55]. However, among patients with cancer admitted to the ICU, those who underwent autologous BMT had the same prognosis as those who did not [19,23]. Benoit and colleagues reported neutropenia to be an independent risk factor for increased mortality in patients with hematologic malignancy admitted to the ICU [16]. However, a subsequent study at the same institution proved this to be no longer valid [56], and based on another large multicenter study in Brazil, Soares et al. recently reported the lack of association between mortality and the presence of neutropenia in patients with cancer [57]. These differences are likely to reflect differences of selection biases by oncologists in providing treatment. The reported prognostic importance of other classic mortality predictors, such as age or characteristics of the malignancy, vary among studies and may mainly depend on ICU admission criteria [18,41]. We recommend not denying ICU admission to elderly patients based on age alone, or to those with advanced malignancies at the earliest phase of the disease, a time when response to therapy is not known.

#### Improved understanding of organ dysfunction

There has been an improved understanding of organ dysfunction, mainly as a result of a close collaboration between hematologists/oncologists and intensivists [24] (Table 2). Clinical experience in managing patients with cancer has led to better understanding of the pathophysiology of acute tumor lysis syndrome [12,46,58,59] and macrophage activation syndrome [14] and to a comprehensive diagnostic strategy of acute respiratory failure [60]. Although the cause-effect relationship cannot be proven, we strongly believe that the improved understanding of organ dysfunction in patients with cancer has translated into better survival.

#### Some groups of patients remain with high and unchanged mortality

In addition to bedridden patients and those with no lifespan expanding therapy (see point 5 following), there are three groups of patients in whom survival rates remain marginally low. These include allogeneic BMT recipients with severe graft-versus-host disease (GVHD) who are unresponsive to immunosuppressive therapy [27,61], patients with multiple organ failure related to delayed ICU admission [10], and specific clinical vignettes in patients with solid tumors, such as pulmonary carcinomatous lymphangitis with acute respiratory failure [62], carcinomatous meningitis with coma [63], or bone involvement by extra-hematopoietic cancerous cells and medullar insufficiency [64].

Several studies have assessed the outcomes of allogeneic BMT recipients admitted to the ICU during the past three decades [61]. Despite careful selection for ICU admission and advances in critical care, the prognosis remains grim, with an overall 1-yr survival rate of less than 10% in patients who receive mechanical ventilation [27,34,65-67]. Outcomes are not related to the source of stem cells (bone marrow vs. peripheral blood vs. cord blood donors), the underlying malignancy for which BMT was performed, and patient-related characteristics, such as age or comorbidities [27,61,67]. Ten factors have been identified to be associated with mortality after a critical care illness--most are surrogate markers of GVHD: 1) BMT from unrelated donor, because of increased risks for GVHD and associated complications; 2) GVHD itself, with epithelial injury and subsequent organ dysfunction (liver, gastrointestinal tract and skin,), and with toxic and infectious complications from immunosuppression (aspergillosis and other severe opportunistic infections); 3) the need for mechanical ventilation (associated with approximately 20% survival); 4) acute respiratory failure and the need for mechanical ventilation 4-6 weeks after BMT (GVHD period, 10% survival); 5) the association of severe sepsis and resistant GVHD; 6) thrombotic microangiopathy due to endothelial activation triggered by GVHD, total body irradiation, toxicity of immunosuppressive regimen, and infectious diseases; 7) multiple organ failure in the setting of severe hepatic veno-occlusive disease; 8) acute respiratory failure from pulmonary aspergillosis; 9) late noninfectious pulmonary complications, including diffuse alveolar hemorrhage [68,69], bronchiolitis obliterans [70], and other new onset obstructive ventilatory disorders; and 10) relapse of the underlying malignancy after BMT. We recommend unrestricted intensive care support of allogeneic BMT recipients in three situations: patients at the earliest phase of transplantation (before GVHD develops) and BMT recipients proposed for ICU admission after 1 year of transplant and without GVHD or with controlled GVHD, and patients who require mechanical ventilation for status epilepticus related to posterior reversible encephalopathy syndrome (PRES). In all other situations, ICU admission and goals of therapy should be decided on an individual basis. Because survival remains exceptional, it seems reasonable to discourage ICU admission and mechanical ventilation in patients with severe sepsis or acute respiratory failure and uncontrolled GVHD.

Patients who develop multiple organ failure are at higher risk for death if their ICU admission is delayed. Khassawneh et al. reported only one survivor in patients admitted with three organ dysfunctions [10]. In patients with multiple myeloma, delayed ICU admission was associated with increased mortality [11]. The nature and the extent of organ dysfunctions, at ICU admission or more significantly after day 3, are good predictors of mortality [17,26,71].

#### Triage criteria that are usually used are unreliable

Marginal survival has been reported in severely impaired or bedridden patients [18,24,30], as well as in patients with no lifespan prolonging anticancer therapy [19]. In these patients, care must be maintained but with transition from cure to comfort. Restricted admission policies based on these two criteria translates into improved survival [23,24]. However, triage criteria for ICU admission remain unreliable. In a prospective study that evaluated the outcomes of patients proposed for ICU admission, 20% of patients who were not admitted because they were considered "too well" died before hospital discharge (mainly after a delayed ICU admission), and 25% of the patients who were not admitted because they were too sick survived [72]. Importantly, this study highlighted the inadequacy of the triage criteria and the need for the development and implementation of new ICU admission policies.

#### At least 3 days of ICU management before making end-of-life decisions (ICU trial)

The use of life-sustaining therapies in most patients with cancer is no longer futile. However, recent data suggest that duration of mechanical ventilation, use of vasopressors, and dialysis are strong predictors of death. For example, marginal survival have been reported in patients who require invasive mechanical ventilation for 3 days or more [17,50]. Studies of patients with neutropenia or septic shock have reported that outcomes were not easily predictable at the time of ICU admission [26]. Identifying patients who remain severely ill, with no improvement (or with worsening condition) after 3 days of full ICU support, may be easier and more effective to appraise outcomes [17,19].

There may be "golden hours or days" of resuscitation associated with improved outcome, for the ICU management of critically ill cancer patients. During this time, everything should be done. Subsequently, the continuation or introduction of life-sustaining therapies in patients whose conditions are worsening may not be beneficial. Further observational studies are needed to confirm the optimal time for the "ICU Trial."

#### Finding a balance between noninvasive treatments and avoiding delays in optimal therapies

Early ICU admission offers the opportunity to use noninvasive diagnostic tests (i.e., sputum analyses instead of bronchoalveolar lavage (BAL) in acute respiratory failure) and noninvasive ventilation (NIV) [48,49]. This noninvasive approach is supported by the lower diagnostic yield of BAL in cancer patients with acute respiratory failure and the easy availability of noninvasive diagnostic tests [40,60]. Although bronchoscopy and BAL can be avoided in a large proportion of patients, those who may benefit should be identified early after admission to the ICU.

#### Close relationship and collaboration need to be developed with hematologists and oncologists to increase the skills of both sides in the overall management of patients with cancer

Undoubtedly, some of the improvement in the outcome of critically ill cancer patients can be attributed to formal and informal free exchange of ideas between intensivists and hemato-oncologists. The hemato-oncologists are able to appraise outcomes and to update the intensivists about therapeutic options and potentials for cure of the underlying malignancies. Hemato-oncologists may teach the key pathophysiological aspects of malignant diseases as well as specific complications. Also, ICU admission decisions should be undertaken by both parties based on the acute medical disease, as well as the underlying disease prognosis and patients' preferences and values. ICU clinicians may be more knowledgeable and experienced in setting goals of life-sustaining therapies based on the reversibility of single organ dysfunction and on the presence of multiple organ failure. Decisions to withhold or withdraw life-sustaining therapies are best undertaken by both parties. Information given to patients' relatives and shared decision-making should be presented by both parties together.

#### Early admission to the ICU for patients with cancer

We are not aware of any study designed to assess the impact of early ICU admission. The following four observations suggest that early admission may improve outcome: a) during the past decade, decreased mortality was observed in association with earlier ICU admission [11]; b) receiving oxygen at a flow higher than 1 liter/min is significantly associated with subsequent need for mechanical ventilation and death [73]; c) performing high-risk procedures in severely ill patients (i.e., bronchoscopy and BAL in hypoxemic patients) may be less harmful if performed in the ICU (sometimes under NIV) [40,51,74,75]; and d) there is a linear relationship between the number of organ dysfunctions and patients' survival, suggesting that patients should best be admitted as early as possible rather than at a time of multiple organ failure where survival remains marginal.

#### Doing everything that can be done, including cancer chemotherapy

When ICU admission is warranted, patients should be treated with a full code status, or according to an ICU trial. In both situations, patients receive everything they need during the first few ICU days and then have their situation reappraised after 3 to 5 days of full ICU support. This full-code status includes the administration of cancer chemotherapy along with ventilatory support, vasoactive agents, renal replacement therapy, and other life-sustaining therapies. Indeed, patients with tumor lysis syndrome, pulmonary or renal infiltration by the malignancy, sepsis related to obstructive pneumonia, or ureteral compression may require life-sustaining therapies until the cancer chemotherapy becomes effective. Studies have shown the feasibility of administering chemotherapy in the ICU, with acceptable short- and long-term outcomes [20,21]. They also have demonstrated that when patients present with severe sepsis or septic shock after recent chemotherapy, outcomes may be better than in those who did not receive recent cancer chemotherapy [21,22].

### Broadening ICU admission policies and clarifying patient's code status at the time of admission

#### ▶ Full code status

**Figure 2:**
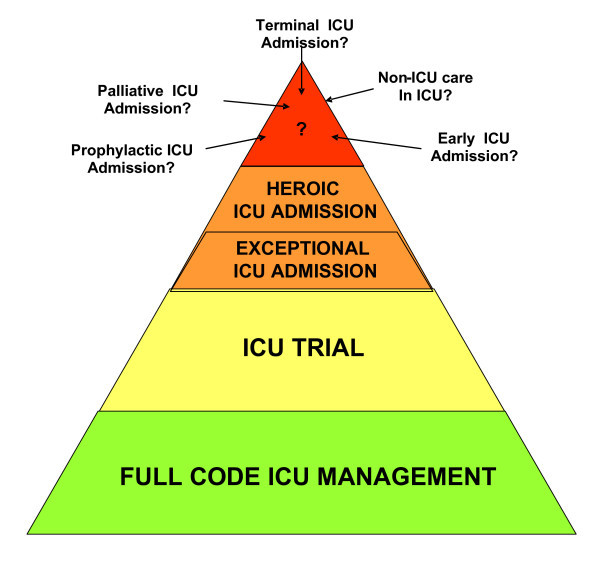
**Alternative to ICU refusal in cancer patients proposed for ICU admission**.

Most critically ill cancer patients are admitted to the ICU during the early management period of aggressive malignancies, i.e., during the first course of chemotherapy (induction or consolidation; Figure 2). Some patients with low-grade hematological malignancies (chronic lymphocytic or myeloid leukemia or low-grade lymphoma) may be admitted at any time of their disease course, mainly for infectious or toxic life-threatening complications. Patients with partial remission (myeloma) or high-risk solid tumors (metastatic breast or ovarian cancer) can be admitted at a later course of their disease, even after several courses of chemotherapy, which provides significant and sustained response with improved long-term survival [2]. All of these patients are admitted to the ICU with a full-code status. The decision-making process is similar to that of other ICU patients without malignancy (Figure 2; Table 3). Also, the need to document patient preferences for resuscitation and end-of-life issues at the time of ICU admission is crucial.

**Table 3:** Different ICU admission policies

Type of ICU admission	Code status	Clinical situation
1. Full code ICU management	Full code	Newly diagnosed malignancies Malignancies in complete remission
2. ICU trial	Unlimited for a limited time period—at least 3 to 5 days	Clinical response to therapy not available or undetermined
3. Exceptional ICU admission	Same as ICU trial	Newly available effective therapy that should be tested in a patient who becomes critically ill
4. Heroic ICU admission	ICU management until conflict resolution	Both hematologists/oncologists and intensivists agree that ICU admission is not appropriate, but patients or relatives disagree with the appropriate level of care
5. Other admission modalities that are performed but not yet formally evaluated		
a) Prophylactic ICU admission	Full code; intensive clinical and biological monitoring; invasive procedures under safer conditions	Earliest phase of high-risk malignancies. Admission to the ICU is warranted to avoid development of organ dysfunction (acute respiratory failure, tumor lysis syndrome, etc.)
b) Early ICU admission	Full code; intensive clinical and biological monitoring; invasive procedures under safe conditions; no life-sustaining therapies	Admission to the ICU in patients with no organ dysfunction but physiological disturbances. ICU is warranted to avoid late ICU admission (condition associated with higher mortality)
c) Palliative ICU admission	Noninvasive strategies only	Admission to the ICU for the purpose of undergoing noninvasive mechanical ventilation as the ceiling of therapy
d) In-ICU non-ICU care	No life-sustaining therapies	Short ICU admission to help for optimal and prompt management (catheter withdrawal, early antibiotics etc.)
e) Terminal ICU admission	No life-sustaining therapies	ICU admission is required to best provide palliative care and symptom control. Controversial issue

#### ▶ ICU trial

In some patients with cancer, the usual ICU admission triage criteria may be unreliable. Nevertheless, establishing the goals of therapy at the time of ICU admission is crucial to optimize their management. The ICU trial is an alternative to ICU refusal in cancer patients [17]. It consists of an unlimited ICU support for a limited time period. Everything is done for at least 3 to 5 days [19]. In a study performed at the Saint-Louis hospital in Paris, France, patients who were non-bedridden and who had a survival prolonging therapy were included [17]. Allogeneic BMT recipients were excluded. Clinicians made clear to patients and families that the ICU trial was an alternative to ICU refusal and that as soon as the situation was considered irreversible with no hope for survival, the level of care was transitioned from cure to comfort. The major result from this ICU trial was that none of the variables available at ICU admission was significantly different between ICU survivors and nonsurvivors. Only after day 3, nonsurvivors had significantly more organ dysfunction than ICU survivors. More recently, we have advocated the use of the ICU trial in patients with newly diagnosed malignancies, but with life expectancy less than 1 year [76]. This study was an attempt to broaden ICU admission policies and to suggest other types of ICU admission to avoid depriving ICU management to patients who may potentially benefit. Indeed, in the evaluation of the ICU trial [17], survival was 20% overall but 40% in patients who were alive and in the ICU after day 3.

#### ▶ Exceptional ICU admission

The first status of admission is the "exceptional ICU" (Figure 2; Table 3). We propose this admission status for patients in whom severe limitation of the performance status are attributable to the malignancy itself and may improve in response to chemotherapy. Another scenario for this status is when evidence emerges from new trials that a new effective therapy is available for a patient without lifespan-prolonging therapy. This does not include results from *in vitro *studies or from phase I/II trials. For example, admission to the ICU for patients with intractable malignancy is not recommended. If the results from a new trial report that using a newly developed drug/protocol allows substantial survival, the question that can be raised is about the benefits from this drug when patients become critically ill. In this case, intensivists and hematologists or oncologists should decide to perform a careful evaluation in five to ten patients. In this circumstance, exceptional ICU admission includes formal discussions between hemato-oncologists and intensivists and must address the nature of ICU management and the time with which a response to therapy can be expected. As for the ICU trial, patients and relatives must agree with ICU admission and fully grasp its objectives.

#### ▶ Heroic ICU admission

A second new status of ICU admission that we sometimes adopt is the "heroic ICU." This ICU management is used to resolve conflicts between ICU clinicians and hemato-oncologists, or between clinicians and patients/relatives about the actual prognosis and the appropriate level of care. The philosophy of this type of ICU admission is that 1 or 2 days of ICU management will make the prognosis more evident to appraise and create mutual trust. Although intensivists are aware that death is the most likely outcome, discussions among the stakeholders may help to resolve the conflicts and mistrust. The use of heroic ICU to resolve conflicts and to develop time for mutual trust between clinicians, intensivists, patients, and relatives may create false hope, raise unrealistic expectations, and can be perceived as highly controversial. We must emphasize that if this status of ICU admission is used more than 2 to 3 times per year, conflict resolution strategies or an attempt to increase the understanding and knowledge about the underlying condition (of the actual prognoses of malignancies or of the outcomes of organ dysfunction in cancer patients) are in order.

#### ▶Other admission policies

**Figure 3:**
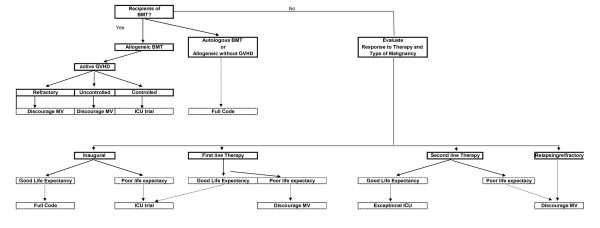
**Code status in cancer patients receiving mechanical ventilation**. Good life expectancy refers to a malignancy where complete remission and long term survival are possible outcomes. Poor life expectancy refers to a malignancy where median life expectancy is below one year.

The common characteristic of all the previous statuses of ICU admission is that patients receive full-code management for an unlimited or a limited time period. Other admission policies need to be evaluated before specific recommendations are made. Such policies include early ICU admission, prophylactic ICU admission, palliative ICU admission, ICU admission for non-ICU care, and terminal ICU admission (Figure 2; Table 3). The terminal admission policy is based on a controversial assumption that the ICU is the best place to die in the hospital and ICU clinicians are skilled to perform adequate palliative care to dying patients. Details on code status and clinical vignettes are reported in Table 3. Figure 3 summarizes our suggestions for ICU admission according to the factors that have been presented above.

### Unanswered questions and research agenda

Many unanswered questions deserve future observational and interventional studies (Table 4). As experts in the management of critically ill cancer patients, we suggest a research agenda to address the following ten burning issues:

**Table 4:** Unanswered questions and research agenda

1) Establishing long-term outcomes in oncology and hematology patients who survive their ICU stay. Do we prolong the dying process or do we actually increase survival?
2) Addressing qualitative outcomes
3) Searching for specific family needs and communication strategies
4) Evaluating new admission policies
5) Improving transition from curative to palliative care
6) Evaluating the impact of the ICU on overall long-term and disease-free survival
7) Defining the appropriate timing for ICU admission (avoiding delays)
8) Appraising prognostic factors of mortality
9) Evaluating outcomes in patients who receive intensive care (e.g., NIV, vasopressor) in the wards
10) Performing qualitative studies before any recommendation on the use of NIV as the ceiling of therapy

1) The first and most intriguing issue is the lack of studies on long-term outcomes in cancer patients who survive their ICU stay. We do not know whether the increase in the number of survival days is only a prolongation of the dying process or if it is an actual increase in survival with good quality of life. Beside ICU and hospital survival, very few studies have addressed survivors' quality of life. For example, in a study of noncancer patients with acute respiratory distress syndrome, survivors lost 18% of their baseline body weight during the ICU stay, experienced severe muscle weakness and fatigue, and had persistent functional disability 1 year after ICU discharge [77-79]. These findings are extremely relevant, because treatment decisions are substantially influenced by the clinical condition of the patient. In critically ill cancer patients, poor performance status has been associated with mortality [18,30]. Moreover, poor performance status may prevent the use of optimally aggressive chemo- and radiation therapy regimens and/or decrease the ability to achieve radical surgical resection, thereby shortening long-term survival. We need studies evaluating outcomes up to 2 years after critical illness. These studies need to investigate survival, treatments that have been implemented, and remission from the malignancy.

2) In addition to physical outcomes, mental health and quality of life outcomes must be assessed in ICU survivors. At this time, no study has specifically evaluated health-related quality of life and post-ICU burden in critically ill cancer patients who survived the ICU. Nelson et al. investigated self-reported symptom experience of cancer patients at the time that they were receiving intensive care support [80]. Most patients reported experiencing pain, discomfort, anxiety, sleep disturbance, or unsatisfied hunger or thirst. Approximately one third reported depression and dyspnea. Significant pain and discomfort were associated with common ICU procedures. Inability to communicate, sleep disruption, and limitations on visiting were particularly stressful among ICU conditions studied. However, no study has assessed the prevalence of these symptoms 6 months or 1 year after ICU discharge. Yet, such assessment is a key issue when addressing the question of ICU admission policies for cancer patients.

3) Studies in relatives of critically ill cancer patients should be developed to seek specific needs and communication strategies. Also, determinants of ICU and post-ICU burden on relatives of ICU survivors should be evaluated, because relatives of critically ill patients may become actual caregivers.

4) In this review, we describe provisional models of ICU admission with various code statuses that have not yet been evaluated. First, criteria for ICU admission need to be described and appraised. Second, new modalities of ICU admission should be evaluated and the balance between their strengths and weaknesses described. Controversial issues, such as palliative or terminal ICU admission, must be discussed at a global level. Indeed, it is possible, yet debatable, that death that occurs in the ICU is perceived as "good" by patients and relatives. However, broadening ICU admission criteria will be obviously associated with increased mortality and associated conflicts [81], clinician's burnout [82], demoralization [83], and further exhausting the limited available resources and ICU beds.

5) One of the most difficult issues in the ICU trial is determining the appropriate time for making end-of-life decisions [84]. Transition from curative to palliative care is complex in cancer patients because of their young age, complex medical conditions, doubts on the actual therapeutic plans, and pressures from consultants and relatives. Implementing new ICU admission policies for cancer patients requires a critical evaluation of end-of-life care that occurs in up to 80% of these patients, i.e., when the irreversibility of the medical condition is deemed certain. Also, quality of dying and death must be specifically assessed in this context. Most importantly, the need to document patient preferences for resuscitation and end-of-life issues at the time of ICU admission is crucial.

6) The ICU benefits for overall long-term and disease-free survival are still unknown. We can hypothesize that patients with cancer who survive the initial complications without residual organ dysfunction will be able to receive full regimen chemotherapy and subsequently more available lifespan-prolonging therapies. However, no ICU study has evaluated the impact of the ICU support on long-term and disease-free survival.

7) Recent studies suggest survival benefits from early admission to the ICU [11]. However, this has never been evaluated properly. Indeed, a randomized, clinical trial designed to admit selected patients with cancer at the earliest phase of the malignancy (before or within first few days of cancer chemotherapy) with only one organ dysfunction may be in order. Besides overall survival, if prevention of organ dysfunction translates into improved outcomes, practical guidelines will be easy to recommend.

8) An appraisal of classic prognostic factors is timely. There are data that suggest that neutropenia and autologous BMT may no longer have prognostic relevance [57]. New determinants of outcome have emerged from recent studies. These new determinants include our ability to make the actual etiological diagnosis rather than treating empirically [40,48,50,51,85], delayed ICU admission [11], cytogenetic data in patients with aggressive malignancies [86], and, clearly, the number and the extent of organ dysfunctions [17,26]. However, additional multicentre cohort studies are needed to identify predictors of death in cancer patients admitted to the ICU, controlling for the ASSESS criteria that we have recently proposed (Table 5) [18]. These include triage criteria for ICU admission, code status implemented at ICU admission, the nature of life-sustaining therapies that are required, and the extent of organ dysfunction at admission, as well as long-term overall and disease-free survival and quality of life.

**Table 5:** ASSESS approach

Domain	Description and rationale
Triage for ICU *Admission*	Triage criteria for ICU admission used by oncologists/hematologists and intensivists. Detailed evaluation of the ICU admission process, including data on non-ICU cancer patients with various levels of organ dysfunction on the wards and data on the effects of early ICU admission
Code *Status*	Code status to be implemented at ICU admission: full code, ICU trial for a short period (3-5 days with full-code status and then reevaluation) or early implementation of palliative care
ICU s*upport *and patient's *evolution*	ICU management, with a reappraisal of the intensity, duration and nature of life-supporting treatments provided in the ICU. Evaluation of incidence, nature and outcome of organ failures and residual organ dysfunction
*Survival*	Beyond short- and medium-term survival by evaluating long-term outcomes (up to 1 year)
Picture of *survivors*	Description of ICU survivors, including qualitative evaluation of the ability to undergo chemotherapy, disease-free survival, functional status, health-related quality of life and post-ICU burden (stress-related disorders, anxiety, and depression)

Adapted from (18)

9) There is an emerging interest for adding NIV to routine supportive care provided in hematology/oncology wards [87-90]. This is partly due to shortage of ICU beds [91] and ICU physicians' reluctance to admit cancer patients with acute respiratory failure [92,93], as well as the unconfirmed assumption that ICU admission may hamper patients' chances of receiving optimal hematology/oncology care and appropriate infection prevention. We advise caution when implementing NIV in hypoxemic patients with cancer [94]. Unless NIV is the ceiling of therapy [95], we believe that NIV should be initiated only in an ICU or high-dependency unit setting, where endotracheal intubation and invasive mechanical ventilation can be safely and timely performed if NIV fails [96].

10) Mortality in patients who receive palliative NIV has been reported in various subgroups of patients [97-101]. Patients with cancer remain poor candidates for palliative NIV, even if some of them may receive some benefit [62]. Additionally, the concept that the ICU is an appropriate setting to deliver palliative or terminal care is highly controversial. However, these studies report no qualitative outcomes, including quality of life, ICU-burden, and quality of dying for the majority of patients who die after NIV. We believe that qualitative studies are mandatory before establishing any recommendation on the use of NIV as the ceiling of therapy in patients with cancer.

## Conclusions

In patients with cancer who require ICU admission, the survival rate has improved. Besides refinements in the selection criteria of patients for ICU admission, advances in hematology and oncology as well as enhancements in ICU management have contributed to this improved survival. In this changing context, clinicians' beliefs regarding the results of ICU management of patients with cancer must be appraised. Also, admission policies must be broadened and closely evaluated to avoid depriving patients who may benefit from life-sustaining therapies.

## Competing interests

The authors declare that they have no competing interests.

## Authors' contributions

All authors contributed to the preparation of this manuscript and critical review of the material.
